# Injuries to the Immature Optic Radiation Show Correlated Thinning of the Macular Ganglion Cell Layer

**DOI:** 10.3389/fneur.2018.00321

**Published:** 2018-05-07

**Authors:** Finn Lennartsson, Maria Nilsson, Olof Flodmark, Lena Jacobson, Jonas Larsson

**Affiliations:** ^1^Department of Neuroradiology, Karolinska University Hospital, Stockholm, Sweden; ^2^Department of Clinical Neuroscience, Karolinska Institutet, Stockholm, Sweden; ^3^Unit of Optometry, Department of Clinical Neuroscience, Karolinska Institutet, Stockholm, Sweden; ^4^Ophthalmology and Vision, Department of Clinical Neuroscience, Karolinska Institutet, Stockholm, Sweden; ^5^Department of Psychology, Royal Holloway, University of London, Egham, United Kingdom

**Keywords:** retrograde trans-synaptic degeneration, fiber tractography, prematurity, retinal ganglion cell layer, optic radiation, visual fields, optical coherence tomography

## Abstract

Injuries to the immature optic radiation (OR) are associated with thinning of the retinal nerve fiber layer and corresponding visual field (VF) defects. The aim of the current study was to seek evidence for causal retrograde trans-synaptic degeneration by exploring the correspondence between the localization and extension of the injury to the OR and the structure of the macular ganglion cell complex, and the relation to VF function. Seven adults (age range 18–35) with visual dysfunction secondary to white-matter damage of immaturity and six healthy adults (age range 22–33) underwent magnetic resonance imaging (MRI). Fiber tractography was used to generate the geniculate projections to the dorsal and ventral striate cortex, delineated by retinotopic functional MRI mapping. The structure of the macular ganglion cell complex was measured with optical coherence tomography. The tractography showed overlaps between the dorsal and ventral geniculo-striate projections. However, in four patients with inferior VF defects, the dorsal projections were to a large extent traversing the space normally solely occupied by ventral projections. This is consistent with structural changes to the OR and suggests of re-organization upon injury. Diffusion parameters were significantly different between patients and controls, and most pronounced in the dorsal geniculo-striate projections, with a pattern indicating primary injury. The macular ganglion cell complex was significantly thinner in the patients and most pronounced in the superior sectors; a pattern particularly evident in the four patients with inferior VF defects. The ratio of the mean thickness of the macular ganglion cell complex in the superior and inferior sectors significantly correlated with the axial and mean diffusivities in the contra- and ipsilateral dorsal striate projections. The results suggest a causal link between injuries to the superior portion of the immature OR and secondary thinning in the macular ganglion cell complex, resulting in inferior VF defects.

## Introduction

Injury to the brain has become the most common cause of visual impairment in children in high-income countries (1). Mitry et al. (2) highlighted a more than twofold increase in the number of new blind and partial-sighted children registered in England between 1982 and 2011. This is as a result of the improved survival rates of critically ill newborns, particularly those born preterm.

Brain lesions acquired during the early third trimester, 24–34 gestational weeks, can be referred to as white-matter damage of immaturity (WMDI) (3), as they either occur in the preterm newborn or *in utero* and most frequently affect to the periventricular white matter (4). The most common forms of WMDI are periventricular leukomalacia followed by germinal matrix hemorrhage, either complicated by intra-ventricular hemorrhage and/or periventricular haemorrhagic infarcts. WMDI has a predilection for the deep white-matter watershed zones (5), especially the peritrigonal area, and can involve the optic radiation (OR). The injury may extend to the lateral geniculate nucleus or the occipital cortex. WMDI is best detected with magnetic resonance imaging (MRI) (6), although subtle lesions may only be detected on diffusion-weighted MRI (dMRI) (7, 8). MRI imaging correlates of WMDI include volume reduction and/or signal changes corresponding to gliosis in these structures (6, 9, 10). Visual impairment due to WMDI is characterized by low or near normal visual acuity, visual field (VF) defects, eye motility problems, and cognitive–perceptual visual problems (11, 12). The inferior VF is commonly affected as bilateral quadrantdysopias/anopias (13), indicating bilateral injuries to the superior portions of the ORs. The severity of MRI changes of WMDI correlates with the degree of cerebral visual impairment (10). However, the exact relationship between the lesion and the OR may be difficult to infer from structural MRI. dMRI and white-matter fiber tractography (14) can visualize the OR (15). The individual streamlines in the OR tract can be color-coded to yield a topographical representation that is then compared with expected neuroanatomy (16). Diffusion parameters can be analyzed along the OR and changes related to tract microstructure (17). In premature neonates, alterations to diffusion parameters along the OR indicate progressive maturation up until term (18) and are related to early visual function (18–20) as well as to the level of white-matter injury (21).

The structure of the retinal nerve fiber layer (RNFL) and the macular ganglion cell complex (including the ganglion cell layer and the inner plexiform layers; GCL_IPL) can be measured with optical coherence tomography (OCT). Thinning of the RNFL and the GCL_IPL can be seen in lesions in the anterior visual pathways (22) and in congenital and later acquired injuries to the posterior visual pathways (23, 24). The latter is suggestive of retrograde trans-synaptic degeneration (RTSD) (25), which has in turn been proven in animal studies (26–28) and in histopathology studies in humans (29). Thinning of the optic tracts (30) and gliotic changes in the lateral geniculate nuclei (31) seen on MRI have been interpreted as RTSD from WMDI, although primary thalamic injuries can also occur in WMDI (32). Studies show topographical correspondences between the thinning of RNFL (23, 33) in cases of homonymous VF defects caused by congenital and acquired injuries to the posterior visual pathways and, similarly, for the GCL_IPL (24, 34) and in later acquired injuries. In a recent study, Rothman et al. (35) showed that, compared to term newborns, very preterm newborns had a thinning of the RNFL in the temporal quadrant and the papillomacular bundle when measured around term equivalent age. They suggested that the RNFL thinning in the very preterm newborns is related to the brain structure abnormalities on MRI at term equivalent age and to the level of general neurodevelopment in children at the age of 18–24 months. The evidence for RNFL thinning and VF defects secondary to OR involvement in WMDI in young adults has recently been illustrated by our research group (36). In this study of seven young adults with visual dysfunction and WMDI, there was, for all cases, a correspondence between the involvement of the superior portion of the OR, assessed by fiber tractography, and thinning of the RNFL in the superior and temporal quadrants. With refined fiber tracking methods and measurements of the macular GCL_IPL structure, which has a direct relation to the VF, a more accurate topographical and structural correlation between the primary injury in the OR and its secondary effects on the GCL_IPL could be studied.

The current study aimed to investigate the topographical and structural relationship between injuries to the OR from WMDI and the macular ganglion cell complex, along with corresponding VF defects.

## Materials and Methods

### Experimental Design

This study used an extended case description design. In a larger cohort of patients with WMDI and visual dysfunctions, collected by Lena Jacobson over 25 years in clinical pediatric ophthalmology, 30 have reached adulthood. These individuals were reviewed by the research group, from memory and their medical records, for their ability to cope with the demanding MRI examination, to perform standardized perimetry and to maintain fixation during OCT examination. After revision, eight individuals were invited to participate in the study (one of whom declined participation). A group of age- and sex-matched controls was recruited.

### Subjects

We investigated seven young adults (five females; age range 18–35 years) with known WMDI, who had presented with visual dysfunction as children. The study is an extension of a previous study (36), and is indexed in the same way as in that study (Subjects A–G). None of the patients have any history of retinopathy of prematurity. In the current study we also recruited six young adults (four females; age range 22–33 years) with no history of prematurity or disturbances of the visual pathways to serve as a reference group.

### Neuroimaging

Neuroimaging was done on a 3-Tesla MR-scanner (Discovery MR750, GE Medical Systems, Milwaukee, WI, USA) using an 8-channel head coil. Conventional MRI included fluid-attenuated inversion recovery images (FLAIR), high-resolution 3-dimensional T1-weighted images (3D-T1w), and, for the patients, axial T2-weighted images. dMRI (twice-refocused SS-EPI, TR/TE = 6,000–7,800/91.6 ms; voxel size, 2.3 mm^3^; ASSET factor 2) were acquired with two baseline images at *b* = 0 s/mm^2^ and 60-gradient directions at *b* = 2,200 s/mm^2^. Two additional images at *b* = 0 s/mm^2^ were collected with reversed phase-encoding polarity to correct for susceptibility-induced image artifacts during post-processing. Retinotopic functional MRI (fMRI) was acquired [GRE-EPI, TR/TE = 1,600/34 ms; flip angle = 65°; resolution 3 mm^3^; ASSET factor 2] with 30 axial slices (interleaved acquisition) covering of the occipital lobe. 154 fMRI-volumes were imaged for a total scan time of 4:06 min. All fMRI datasets were, prior to any analysis, motion-corrected and realigned using the rigid-body registration tool FLIRT within the FMRIB Software Library (FSL, version 5) (37).

### Definition of the Primary Visual Area

Area V1 was defined in individual subjects using standard retinotopic fMRI mapping methods (38, 39). Stimuli consisted of contrast-reversing checker-boards shown within counter-clockwise/clockwise rotating wedge apertures (subtending a polar angle of 22.5°) and contracting/expanding ring apertures (subtending a visual angle of 0.75°), presented against a uniform gray background. Maximum stimulus eccentricity was 11°. The visual stimuli were presented to each subjects’ dominant eye (the non-dominant eye was covered with an eye patch) using an fMRI-compatible visual stimulus system (NordicNeuroLab, Bergen, Norway). The complete fMRI-stimulus sequence consisted of two blocks of three complete stimulus cycles (each 16*TR seconds), interspersed within three blocks of non-stimulation (blank gray screen), each lasting one cycle (16*TR seconds). The first non-active block was preceded by 10 “dummy scans” for a total stimulus time of 4:06 min. By shifting the counter-clockwise wedges and contracting rings stimuli three time frames back and averaging with the corresponding clockwise wedge and expanding rings time-series, a coarse compensation for the hemodynamic response was obtained (38). The rationale for including non-active blocks was to provide a baseline for estimation of population receptive fields. However, in this study the fMRI volumes for these time points were excised from the time-series, and only the active stimulus data were used for the analysis.

**Figure 1:**
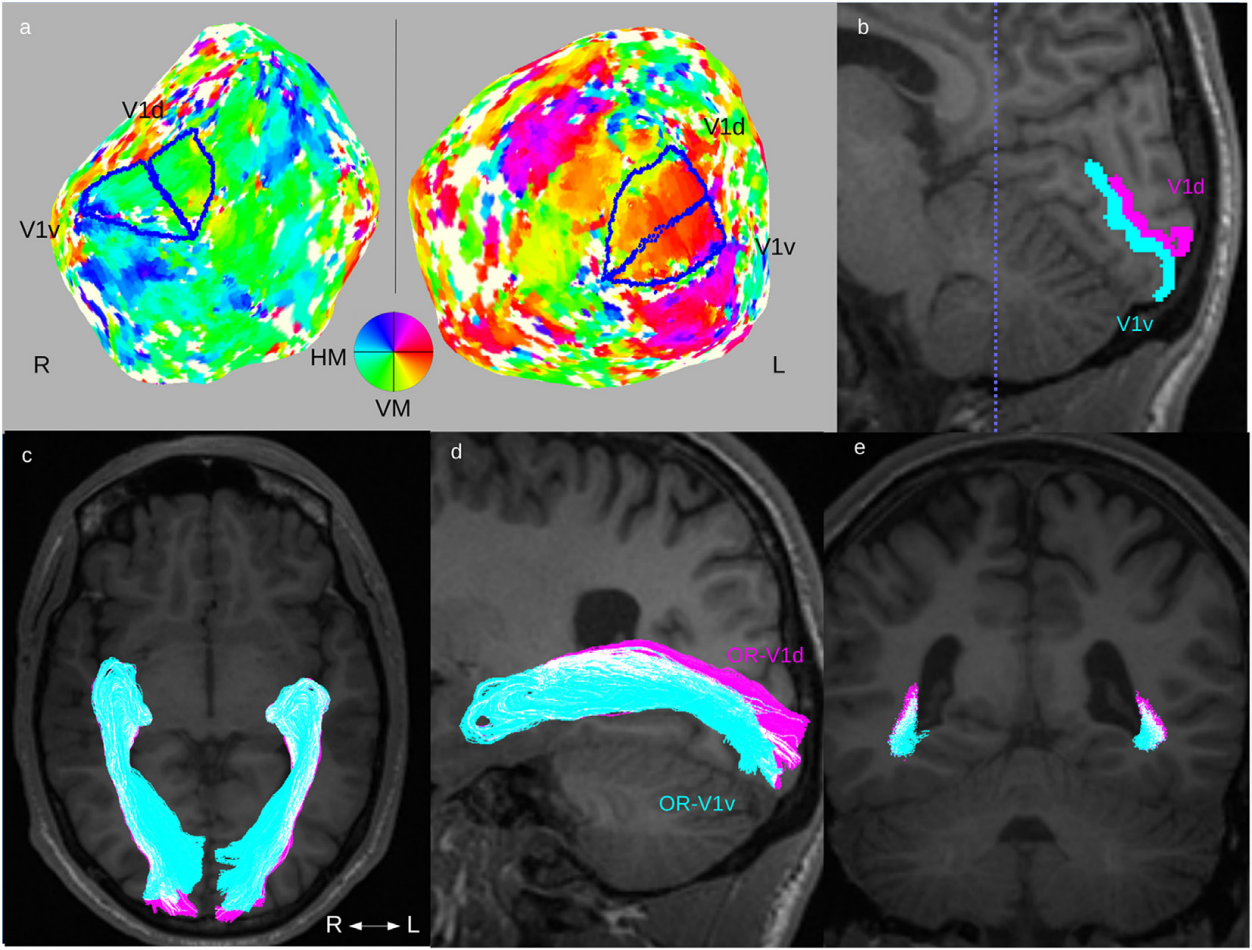
Fiber tractography of the optic radiation (OR). Overview of the complete fiber tracking procedure of the OR illustrated in one patient (Subject F). This patient had normal OR on fiber tractography and normal GCL_IPL thickness on OCT, and no visual field defects. Upper row: **(A)** definition of V1d- and V1v-ROIs from the retinotopic fMRI activation maps on cortical flattened 3D-T1w maps. **(B)** The left side’s V1d- (pink) and V1v-ROI (turquoise) mapped on a 3D-T1w image in sagittal midline section around the calcarine sulcus. Lower row: fiber tracts for OR-V1d (pink) and OR-V1v (turquoise) overlaid on 3D-T1w image in **(C)** an axial, **(D)** a parasagittal (only tracts on left side), and **(E)** a coronal section at the level of the trigone [dotted blue line in **(B)**]. Note the spatial overlap between the OR-V1d and OR-V1v tracts along their course. Abbreviations: R, right; L, left; H/VM, horizontal/vertical meridian.

The retinotopic activation maps were visualized on flattened cortical surfaces from the 3D-T1w dataset using custom Matlab software mrLoadRet.^1^ The cortical flattening was performed using the SurfRelax software package^2^ (40). The procedure includes segmentation of gray/white-matter boundaries, which, in the patient group, were commonly compromised by the WMDI lesions, and this step was, therefore, manually edited in many patients. The V1-area was defined on the phase map from the polar angle stimulus (39). The border between its ventral and dorsal parts, V1v and V1d, were defined as the boundary corresponding to the mean phase of V1 (representing the horizontal meridian). Examples are seen in Figure 1A. The V1d- and V1v-ROIs were then mapped into diffusion space, as illustrated in Figure 1B. The manual editing of the gray/white-matter segmentation maps and the delineation of the V1d- and V1v-ROIs were done by an experienced neuroradiologist (Finn Lennartsson).

### Fiber Tractography of the OR

The dMRI data were corrected for motion-, susceptibility artifacts, and eddy-current induced distortions using the eddy tool in FSL (41), and co-registered to the 3D-T1w dataset with FLIRT in FSL (37). Estimation of diffusion tensors and fiber orientation distributions (FOD) with constrained spherical deconvolution (42) was done for every voxel. Probabilistic streamline fiber tractography of the OR to the V1d-ROI (OR-V1d) and the V1v-ROI (OR-V1v) was performed using the MRtrix package (J-D Tournier, Brain Research Institute, Melbourne, Australia^3^) (43) by seeding streamlines in a 4-mm sphere encapsulating the lateral geniculate nuclei (36, 44) and propagating the streamline through the FOD-field until it entered the ipsilateral V1d- or V1v-ROI (stop criteria). The placement of the seeding spheres was done by an experienced neuroradiologist (Finn Lennartsson), which for the patients were chosen to be equal to the locations in the previous study (36). The rater was not blinded to the VF maps of the patients, as these had been presented in the previous study, however, neither VF maps nor OCT measurements were consulted during the analysis. To reduce false positive streamlines, exclusion ROIs for corpus callosum, the anterior commissure and the immediate medial and dorsal aspects to the lateral geniculate nuclei were used. The tracking was terminated when 5,000 accepted streamlines had been generated for each of the OR-V1d and OR-V1v tracts. The OR-V1 was defined as the entire set of 10,000 streamlines in both OR-V1d and OR-V1v tracts. To reduce connections of low probability (typically false positives) streamlines that entered voxels with a visitation count <5 (<10 in OR-V1) were rejected (i.e., 0.1% of total streamline count). This resulted in an average rejection of 8% (range 5–11%) of streamlines, for both controls and patients. The remaining set of streamlines was considered to be the desired tracts. The fiber tracking procedure is outlined in Figure 1. For all tracts, tract density images (45) were created to serve as probability maps.

The OR tracts were visually assessed according to their expected topographical distribution (16), with emphasis on OR-V1d and OR-V1v, which were assumed to contain fibers representing the lower and upper VF, respectively (46). The spatial overlap between the OR-V1d and OR-V1v tracts was evaluated using the Cohen’s kappa coefficient (47, 48) of the corresponding probability maps (binarized). Diffusion tensor (DTI) parameters of fractional anisotropy (FA), mean diffusivity (MD), axial diffusivity (D_//_ = largest eigenvalue), and radial diffusivity (D_⊥_ = mean of second and third eigenvalues) were calculated along each streamline, then averaged for each tract and used for statistical comparison. Apparent fiber density (AFD) (49) connectivity (afdconnectivity command in MRtrix3; see text footnote 3) was calculated for each tract, based on the fact that the integral over an FOD-lobe is proportional to the volume of the underlying MR tissue signal (intra-cellular at higher *b*-values) along this direction (49). By summing the integrals of the FODs traversed by all streamlines, a total tract volume is obtained. This tract volume can then be divided with the average streamline length, giving an average transverse area of the tract, which then corresponds to a measure of the connectivity of the tract, independent of its length. The AFD connectivity measure for each OR tract was used for statistical comparison.

### Optical Coherence Tomography of the Macular Ganglion Cell Complex

Retinal measurements were obtained by the use of the spectral domain OCT from the Carl Zeiss model HD-Cirrus OCT™ 5000 (Carl Zeiss Meditec, Dublin, CA, USA). Pupillary dilation was not needed.

The macular cube scan 512 × 128, covering 6 mm × 6 mm of the retina with the fovea centered, was used for imaging the macular structure. The perifoveal ganglion cell layer thickness was estimated by analyzing the GCL_IPL complex as performed automatically by the OCT software program (Ganglion Cell Analysis). The GCL_IPL complex was calculated in an elliptic annulus centered on the fovea with a vertical inner radius and vertical outer radius of 0.5 and 2.0 mm, respectively, and a horizontal inner radius and horizontal outer radius of 0.6 and 2.4 mm, respectively (50). The GCL_IPL thickness values were obtained from six sectors (superior, superonasal, inferonasal, inferior, inferotemporal, and superotemporal), representing a superior and an inferior hemisphere of the macular area. Based on the assumption that the superior GCL_IPL would be more affected in WMDI, the ratio between corresponding superior and inferior sectors and their means was calculated and used for statistical comparison. The ratio of the mean values of the superior and inferior sectors was chosen to correlate with the calculations of tract parameters in the ipsi- and contralateral OR.

Only data from OCT measurements with high image quality were used. High image quality of the OCT measurements was defined as images with small or negligible influence by eye movements and/or blinking, and signal strength of 6 or higher.

### VF Examination

All patients had undergone VF examination using the standard automated perimetry Humphrey Field Analyzer. The SITA Fast 24-2 program was used in order to quantify and map the VF function. Pattern deviation plots, highlighting the localized loss due to neurological damage and filtering out generalized loss, was used for comparison between the superior and inferior VF.

### Statistical Analysis

Statistical analysis was done using the Statistics Toolbox in Matlab (version R2015a; MathWorks, Natick, MA, USA).

The non-parametric Wilcoxon signed-rank test was used to assess left-right side-differences for tract parameters in OR tracts and the spatial overlap between the OR-V1d and OR-V1v tracts within each group. The same test was used to explore differences within each group of the OCT measurements of GCL_IPL thickness for all sectors between both eyes, and between the superior and inferior sectors in each eye. The non-parametric Wilcoxon rank-sum test (Mann–Whitney *U*-test) was used to assess differences between patients and controls for tract parameters, spatial overlap and OCT measurements.

Associations between the mean value of the left and right tract parameters and the mean value of the left and right eye’s ratio of the averaged GCL_IPL thickness in the superior and inferior sectors were explored by calculating the non-parametric linear correlation coefficient (Spearman’s rho). A more realistic model was also explored by inferring associations between the GCL_IPL thickness for each eye and the tract parameters in the ipsi- and contralateral OR tracts using a linear regression model:

$${\text{GCL\_IPL}}_{\text{ipsilateral}}=\alpha +\beta \cdot {\text{tract\_parameter}}_{\text{ipsilateral}}+\gamma \cdot {\text{tract\_parameter}}_{\text{contralateral}}$$

The GCL_IPL measurements contrasting the superior and inferior visual hemispheres could then be linked with tract parameters from both hemispheres as assumed from anatomy due to the midline crossing of the nasal retinal fibers in the optic chiasm and uncrossed temporal retinal fibers.

All statistical inference was done with two-tailed tests and *p* < 0.05 as significance level.

## Results

### Retinotopic Delineation of V1

Cortical activation maps of V1 could be defined in both hemispheres in all controls and in all patients. The cortical activation maps did not on visual inspection differ between patients and controls. All the mapped V1-ROIs showed the expected location in the medial occipital cortex, with their dorsal and ventral subparts largely localized on either side of the calcarine sulcus. The results indicate that the locations of V1 in individuals with WMDI do not differ from that in controls (Figures 1A,B), and are in line with a recent case study on WMDI using retinotopic fMRI mapping (51).

### Fiber Tractography of the OR

**Figure 2:**
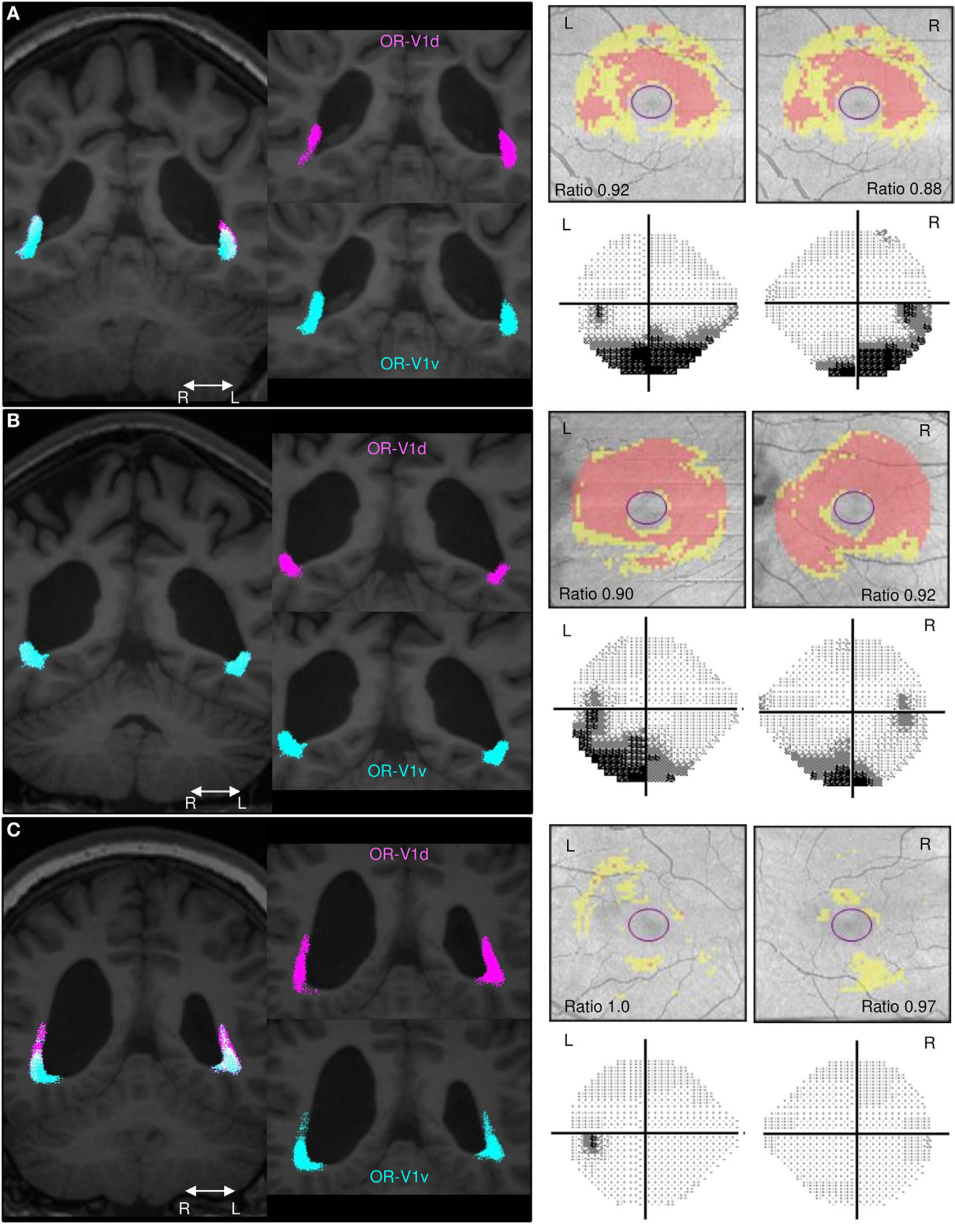
Comparison of the spatial distribution of streamlines in the OR, the GCL_IPL measurements and the VF maps. Spatial distribution of streamlines in the OR-V1d (pink) and OR-V1v (turquoise) tracts for patients. **(A)** Subject A, **(B)** Subject C, and **(C)** Subject G and their corresponding GCL_IPL measurements and visual field (VF) maps. Left column: tracts overlaid on 3D-T1w images in a coronal section at the level of the trigone. Right column: maps of the macular GCL_IPL thickness topography with yellow indicating minor thinning and red more severe thinning. The ratios of the average GCL_IPL thickness in superior and inferior sectors are inserted. VF maps assessed with Humphrey field analyser. The grayscale runs from white to black, with more intense darkness illustrating deeper sensitivity reduction. Grayscale printouts illustrate the reduced sensitivity in the inferior hemifield, typical for this study group. Note the topographical correspondence between the spatial displacement of the OR-V1d tract into the space occupied by the OR-VRv tract in **(A)** Subject A and **(B)** Subject C and the relative thinning of superior sectors of the GCL_IPL and inferior VF defects. This is not the case in **(C)** Subject G. Despite having a large white-matter damage of immaturity [bilateral intra-ventricular hemorrhage (L > R) and right-sided periventricular hemorrhagic infarct (36)], the primary visual system is not affected in this patient. Abbreviations: R, right; L, left.

The ORs were reliably reconstructed in all controls and patients. In both the left and the right hemispheres, OR-V1d and OR-V1v showed a substantial overlap in all controls and patients Figure 1 and Figure 2). This consisted, first, of a large overlap of the majority distribution of the streamlines and, second, of the presence of a substantial number of streamlines in the opposite location, i.e., streamlines in the anterior (Meyer’s) loop that terminate in the V1d-ROI and streamlines in the posterior loop that terminate in the V1v-ROI. The results imply that in probabilistic fiber tractography of the OR with cortical targets a topographical spread in the streamline distribution along OR can be expected, but that the general pattern follows the expected neuroanatomy (46).

A similar topographical pattern to that in controls was seen in three patients (Subjects E–G; example in Figure 2C), whereas four patients (Subjects A–D; examples in Figures 2A,B) showed a deviating pattern with the OR-V1d streamlines more or less encapsulated within the OR-V1v tract during their periventricular course. This indicates that the OR-V1d is more affected by injury with displacement to regions of white matter normally solely occupied by the OR-V1v.

### Comparison of Tract Parameters in the OR

There were no significant differences among the controls in DTI parameters, AFD connectivity, or the spatial overlap between OR-V1d and OR-V1v between the left and right OR tracts.

Among the patients, there were no differences in DTI parameters, nor the spatial overlap between OR-V1d and OR-V1v, the between the left and right OR tracts. There was, however, a significant inter-hemispheric difference in AFD connectivity in OR-V1 and OR-V1d, with AFD connectivity being lower on the left side for all patients. The difference was slightly larger in OR-V1d.

Compared to controls, patients showed significant increases in MD, D_//_, and D_⊥_ with congruent decreases in FA in all OR tracts. The differences were most pronounced for OR-V1d. The AFD connectivity was significantly lower in all tracts in patients than in controls and the spatial overlap of OR-V1d and OR-V1v was significantly higher in patients than in controls.

**Figure 3:**
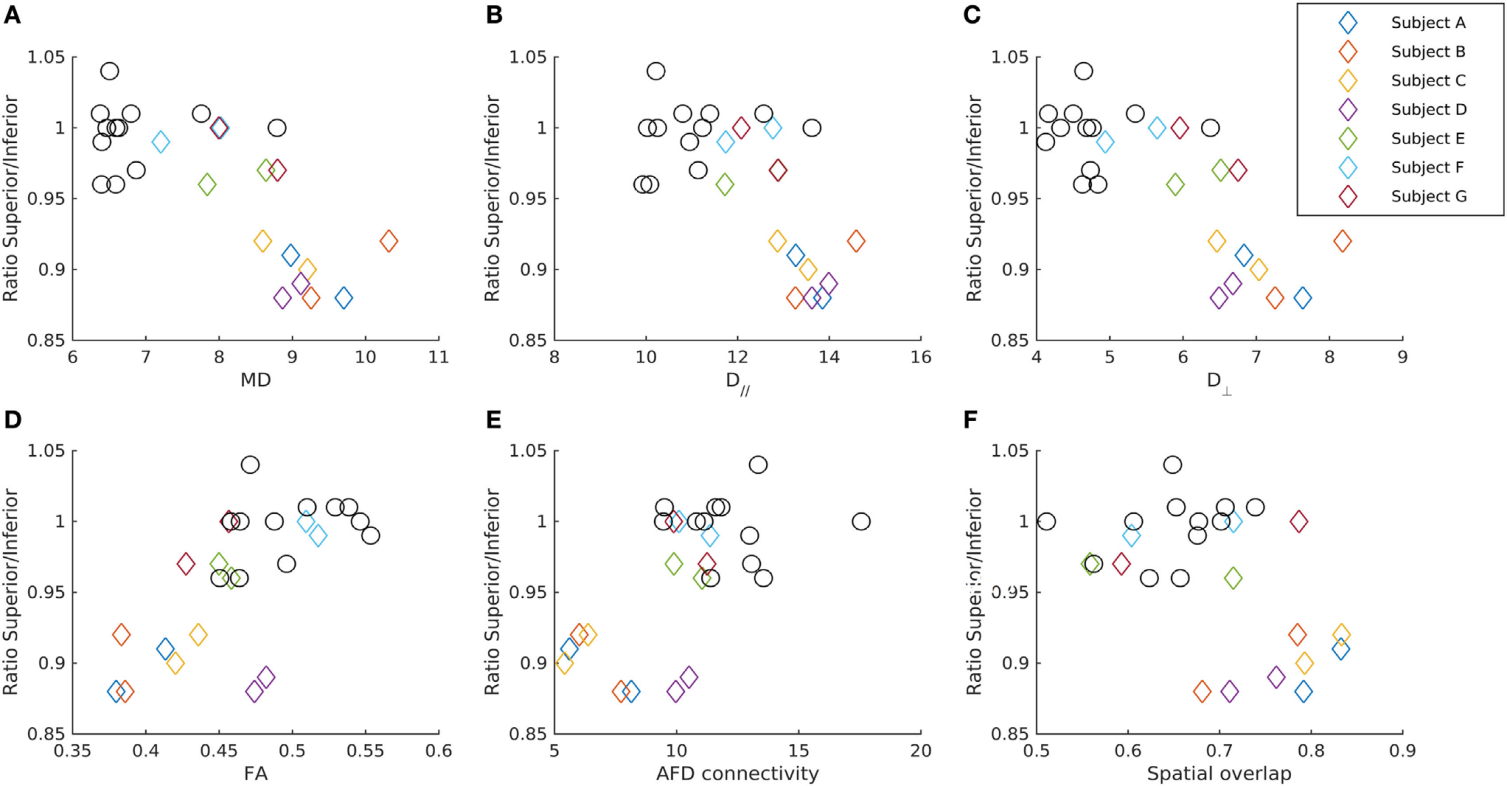
Relationship between the GCL_IPL thickness and diffusion parameters in the OR-V1d tracts. The ratio of the average GCL_IPL thickness in superior and inferior sectors in each eye for patients (diamonds) and controls (circles) plotted against **(A)** mean diffusivity MD, **(B)** axial diffusivity (D_//_), **(C)** radial diffusivity (D_⊥_), **(D)** fractional anisotropy (FA), and **(E)** apparent fiber density (AFD) connectivity in the ipsilateral OR-V1d tract, as well as against the **(F)** spatial overlap between the ipsilateral OR-V1d and OR-V1v tracts. Data are color-coded for each patient (Subjects A–G). Diffusivity in unit 10^−4^ mm^2^/s. AFD connectivity in arbitrary unit. Spatial overlap given as the Cohen’s kappa coefficient (47).

An overview of the tract parameters for controls and patients are listed in Table 1. All tract parameters for all controls and patients in OR-V1d are plotted in Figure 3.

**Table 1:** Tract parameters and the spatial overlap for the OR tracts.

	Side	Tract	FA	MD	D_//_	D_⊥_	AFD connectivity	Spatial overlap
Controls	Left	OR-V1d	0.49 (0.46–0.54)	6.65 (6.38–8.79)	10.97 (10.03–13.62)	4.66 (4.16–6.38)	11.36 (9.46–13.34)	0.69 (0.56–0.74)
		OR-V1	0.49 (0.46–0.55)	6.72 (6.50–9.05)	11.16 (10.04–14.00)	4.70 (4.21–6.57)	12.02 (10.46–14.59)	
		OR-V1v	0.49 (0.44–0.55)	6.79 (6.58–9.29)	11.32 (10.04–14.34)	4.74 (4.27–6.77)	11.00 (8.89–13.83)	

	Right	OR-V1d	0.49 (0.45–0.55)	6.59 (6.39–7.76)	10.60 (9.93–12.57)	4.69 (4.13–5.35)	12.41 (10.81–17.56)	0.64 (0.51–0.68)
		OR-V1	0.49 (0.46–0.56)	6.63 (6.48–8.04)	10.79 (10.17–13.03)	4.72 (4.15–5.54)	13.67 (12.54–19.37)	
		OR-V1v	0.50 (0.47–0.56)	6.67 (6.56–8.29)	10.98 (10.41–13.44)	4.73 (4.18–5.71)	12.41 (10.46–17.49)	

Patients	Left	OR-V1d	0.45 (0.38–0.51)^b^	8.87 (8.00–10.32)^b^	13.27 (12.08–14.59)^b^	6.52 (5.65–8.18)^b^	9.88 (5.41–10.09)^a^^,^^b^	0.79 (0.56–0.83)^b^
		OR-V1	0.45 (0.39–0.51)^b^	8.92 (8.03–10.29)^b^	13.54 (12.10–14.65)^b^	6.76 (5.90–8.11)^b^	10.34 (5.83–11.18)^a^^,^^b^	
		OR-V1v	0.45 (0.39–0.52)^b^	8.70 (8.06–10.49)^b^	13.80 (12.11–15.52)^b^	6.49 (6.04–8.04)^b^	7.67 (5.47–10.35)^b^	

	Right	OR-V1d	0.44 (0.38–0.52)^b^	8.80 (7.21–9.70)^b^	12.88 (11.72–13.99)^b^	6.68 (4.94–7.63)^b^	10.51 (6.38–11.36)^a^^,^^b^	0.71 (0.59–0.83)^b^
		OR-V1	0.44 (0.39–0.53)^b^	8.68 (7.33–9.61)^b^	12.95 (12.04–14.11)^b^	6.50 (4.98–7.48)^b^	11.07 (6.79–13.18)^a^^,^^b^	
		OR-V1v	0.45 (0.40–0.53)^b^	8.64 (7.45–9.92)^b^	13.03 (12.29–14.60)^b^	6.44 (5.02–7.58)^b^	9.01 (6.57–11.48)^b^	

*Measurements by side (left/right), summarized as median and range (min–max) for all controls and all patients in the different OR tracts. OR tracts are indexed as projections to the V1-area or its part dorsal part above (V1d) and ventral part below (V1v) the horizontal meridian. FA, fractional anisotropy; MD, mean diffusivity; D_//_, axial diffusivity; D_⊥_, radial diffusivity; AFD, apparent fiber density. Diffusivity expressed as 10^−4^ mm^2^/s. AFD connectivity in arbitrary unit. Spatial overlap given as the Cohen’s kappa coefficient (47)*.

*^a^Denotes significant left–right side-differences among the controls or the patients using the non-parametric Wilcoxon signed rank test*.

*^b^Denotes significant difference of the tract measurements (both sides pooled) between controls and patients using the non-parametric Wilcoxon rank sum test (Mann–Whitney *U*-test). Statistical inference was done with two-tailed tests and *p* < 0.05 as significance level*.

### Optical Coherence Tomography of the Macular Ganglion Cell Complex

There were no differences among controls in the GCL_IPL thickness between the eyes, nor within each eye.

Among patients, there were no differences in the GCL_IPL thickness between their eyes. The average GCL_IPL thickness of the superior sectors was significantly thinner than that of the inferior sectors, including the ratio of the averaged values.

The average GCL_IPL thickness in the superior and inferior sectors, including the ratio of the averaged values of the superior and inferior sectors, were thinner in patients compared to controls.

Interestingly, the four patients (Subjects A–D) with inferior VF defects (36) had significantly lower ratios of the averaged values in superior and inferior sectors compared to the three patients (Subjects E–G) without VF defects. Indeed, two of these (Subjects F and G) had GCL_IPL thickness values that were within the normal range.

The OCT measurements of the GCL_IPL thickness are listed in Table 2, and the ratio of averaged superior and inferior measurements are illustrated in Figure 3.

**Table 2:** The GCL_ILP thickness in the eyes of patient and controls.

Subject/sex/year born	Side	Average in superior sectors	Average in inferior sectors	Ratio of averages in superior and inferior sectors
Controls	Left	80.35 (70–97)^a^	79.85 (70.3–96)^a^	1.005 (0.97–1.04)^a^
	Right	80.5 (68.7–94.3)^a^	82 (71.7–95.3)^a^	0.995 (0.96–1.01)^a^
A/M/1977	Left	55.7	61.3	0.91
	Right	54	61.7	0.88
B/F/1993	Left	68.7	74.7	0.92
	Right	64.3	73	0.88
C/F/1989	Left	60.7	67.7	0.9
	Right	62	67.3	0.92
D/F/1988	Left	62.7	71.3	0.88
	Right	67.3	75.3	0.89
E/F/1985	Left	62.7	64.7	0.97
	Right	65.7	68.7	0.96
G/F/1995	Left	78.3	78	1
	Right	77.3	80	0.97
F/M/1991	Left	83.7	83.3	1
	Right	80	81	0.99

*The GCL_ILP thickness (in micrometers) is given as the mean value of the measurements in the superior and inferior sectors for each eye and as their corresponding ratio. This is indicated by side (left/right) for each patient (Subjects A–G) and as the range in controls [median (min–max)]*.

*^a^Denotes significant difference of the OCT measurements (both sides pooled) between controls and patients using the non-parametric Wilcoxon rank sum test (Mann–Whitney *U*-test). Statistical inference were done with two-tailed tests and *p* < 0.05 as significance level*.

### Associations Between the OR and the Macular Ganglion Cell Complex

For the seven patients, there were significant negative correlations between the mean of the left and right eye’s ratio of the averaged GCL_IPL thickness in the superior and inferior sectors and the left-right tract average of MD and D_//_ in the OR-V1 and OR-V1d tracts (Figures 2 and 3). No correlations were found for the OR-V1v tract, or between any of the tract and OCT measurements for the controls.

For the linear regression model, there was a significant negative correlation between the ipsi- and contralateral D_//_ (α = 1.73, β = −284, γ = −326, *p* < 0.005, *R*^2^ = 0.837) and MD (α = 1.42, β = −273, γ = −282, *p* < 0.05, *R*^2^ = 0.70) in OR-V1d tracts and the ratio of the averaged GCL_IPL thickness in the superior and inferior sectors in the ipsilateral eye (Figures 2 and 3) in the seven patients. Hence, a large amount (84%) of the variation in the ratio of the superior and inferior GCL_IPL thickness could be explained by a negative linear correlation with the D_//_ in the ipsi- and contralateral OR-V1d. Correlations were also found for these parameters in the OR-V1 tracts, with almost identical coefficients and slightly lower significance levels, however, without reaching significance for MD (*p* = 0.064). A joint linear model with MD and D_//_ did not explain any further variation, which is to be expected, as the parameters need to be inter-correlated. The interpretation is that a larger D_//_, driving an increase in MD, in OR-V1d is indicative of injury and is correlated with the ratio of the averaged GCL_IPL thickness in the superior and inferior sectors in the eye and the occurrence of inferior VF defects.

## Discussion

This study shows a correlation between the severity of injury to the OR in WMDI and secondary neurodegeneration in the macular ganglion cell complex. The study extends previous results in the same cohort of a correspondence between injuries to the retro-geniculate visual pathways and thinning of the RNFL. In the current study, we used retinotopic fMRI mapping to delineate the dorsal and ventral V1-activation maps and use these as input to the fiber tractography of the OR to achieve a separation of the geniculo-striate connections above (the OR-V1d tract) and below (the OR-V1v tract) the horizontal meridian. The OCT similarly provided us with a separation of the GCL_IPL measurements above and below the horizontal meridian. With this we could convincingly show that there is, indeed, a strict topographical and quantitative relationship between the injury in the superior portion of the OR, evaluated in the OR-V1d tract, and thinning of the GCL_IPL above the horizontal meridian. Injuries extended, to a lesser degree, into the inferior OR, evaluated in the OR-V1v tract, with concomitant thinning in the inferior GCL_IPL, but did not correlate with the characteristic macular findings. The results are compelling and consistent with the extent of the VF defects, and provide strong evidence of RTSD in the immature visual system.

### Accuracy of the OR Tracts

In order to evaluate the topographic relationship between the extent and location of the OR damage and its relation to retinal ganglion cell atrophy, we used probabilistic fiber tractography. Although, the study rater Finn Lennartsson was not completely blinded to the VF maps during the analysis, as the VF maps had been presented in the previous study, care was taken to objectify the tracking procedure by, first, using the same location for the seeding spheres in the lateral geniculate nuclei as in the previous study and, second, using the cortical retinotopic activation maps as tracking targets. The V1-ROIs were defined, using standard procedures (38, 39), to include all reliable activation belonging to V1 on the retinotopic VF maps. All patients did show reliable activation in the cortical field maps which in turn did not visually differ from the corresponding maps in controls. It is, therefore, believed that the V1-ROIs captured the true retinotopic fMRI activation in V1 for all participants, despite any preconception of the VF function. The objective of using probabilistic fiber tractography in the current study to generate streamlines in the OR that, indeed, reached the dorsal and ventral V1-area (the OR-V1d and OR-V1v tracts) in all participants could hereby be guaranteed.

When opting for a probabilistic tracking algorithm, a degree of spatial spread of the streamlines of OR-V1d and OR-V1v was expected, especially since the seeding and waypoint ROIs were far apart. However, surprisingly, a rather substantial overlap was seen (Figures 1 and 2), including on the thresholded probability maps (data not shown). Previous studies that aimed to visualize the topographical distribution in the OR-V1 (44, 52, 53) appear to suggest a more precise distinction of the borders, but none of these studies provided quantitative measures of this spread, and it is possible the discrepancy reflects how data were visualized. The evidence of a spatial distribution in the OR was supported in our study by the finding that patients with extensive injuries had a large spatial overlap between OR-V1d and OR-V1v, with OR-V1d confined to the space occupied by OR-V1v (Figures 2A,B). These patients (Subjects A–D) had more extensive inferior VF defects and abnormally low ratio of the averaged GCL_IPL thickness in the superior and inferior sectors, but showed activation in V1d-area on retinotopic fMRI mapping. Streamlines in OR-V1d are connections to the V1d-ROI, and must, from a probabilistic fiber tracking perspective, represent the most plausible connections given the dMRI data. Similar compression/dislocation of tracts has been seen in the motor system in lesion-affected areas in patients with unilateral cerebral palsy (54) and in a recent case study on WMDI (51). The displacement OR-V1d tract could imply compensatory changes to the OR in WMDI (55); however, we cannot say whether this is the strengthening of already existing connections or recruitment of new fibers. Both are possible as WMDI occur during the time frame of the developing connectivity (56), and events of plasticity might take place. Suggested by the topography of the OR-V1d and OR-V1v, this may be from the developmental mechanism of preserving the cortical maps (57). A comparison with patients suffering from lately acquired injuries to the OR rendering, e.g., homonymous quadrantanopias, could answer this.

### Tract Parameters Along the OR

The pattern of change in DTI parameters in our study suggests primary injuries to the OR (17). Throughout, averaged values along OR were used. WMDI commonly engaged a large part of the OR-V1, especially in the most affected patients (Figure 2), and extracting averaged tract values is, therefore, likely to be representative. The amplitude of the FOD-lobe along a certain direction reflects the dMRI-signal of that specific fiber population and is proportional to the underlying AFD (49). In the posterior periventricular white matter the FOD-lobes in the direction of OR show the largest amplitude (58). Hence, the OR is the dominating fiber population and the DTI model would, therefore, primarily, reflect the OR. Our interpretation is that the DTI changes seen reflect a general loss of structural coherence in the voxels traversed by the OR due to WMDI, and that this is most aggravated in OR-V1d. The concurrent, topographically corresponding, and correlating thinning of GCL_IPL hereby confirms RTSD in the visual system in WMDI.

The AFD connectivity measure, representing the average transverse tract area, was used to study the strength of connectivity of the geniculo-striate connections, in order to encapsulate both the macroscopic structure of the tract and the microscopic entities of the FODs. Our study showed lower connectivity in patients compared to controls, particularly so in three patients (Subjects A–C; Figure 3) with evidence of RTSD, are congruent with the changes to the DTI parameters (Figure 3), and are most pronounced in OR-V1d tract. The interpretation of the lower AFD connectivity is a decrease in the average tract area due to a reduction in the number of geniculo-striate afferents after injury. These results are both in line with previous work showing that AFD is a sensitive marker for specific tract injury in unilateral cerebral palsy due to WMDI (59) and with the AFD changes seen in the OR in glaucoma patients (60). Although our study did not use a common constrained spherical deconvolution response function, as required for strict cross-subject comparisons (49), the difference in result is assumed to be minor as all participants’ response functions showed very similar parameters (estimates of spherical harmonics coefficients) (42), and no systematic differences were seen between patients and controls. The decrease in AFD connectivity indicates that more advanced dMRI methods can be explored to better probe the underlying pathophysiological changes upon WMDI. This would help us to understand the morphological changes seen to the OR-V1d tracts in the patients with evidence of RTSD in this study.

The key here is that we could show that the findings in the OR-V1d tracts correlated with the macular findings, whereas the OR-V1v tracts did not. A result that would not have been possible to obtain in the previous study as we were then only able to generate the entire OR.

### Optical Coherence Tomography Measurement of the Ganglion Cell Complex

The secondary GCL_IPL thinning correlated with the severity of the primary injury in the OR-V1d. This makes the system ideal for studying RTSD, and can be done in a clinical setting. However, the GCL_IPL thinning has shown a progression in atrophy during the first years after later acquired injuries (61). Similarly, a delay could be expected in early brain lesions, which is why the optimal timing of OCT ought to be investigated further.

The clear topographical correlation between the thinning of superior sectors in the GCL_IPL and the inferior VF defects is compelling. As expected, the pattern of GCL_IPL thinning was more predictive of VF defects compared to the tract parameters. This illustrates that the macular GCL_IPL has a more direct correspondence with the (central) VF than the RNFL. The RNFL is often reported and analyzed in four quadrants not respecting the vertical and horizontal midlines, which is typical for neurological VF damage in this patient group. The previous study on this cohort showed thinning of the superior part of the peripapillary RNFL (36), which correlated with injury involvement of the superior portion of the OR. However, at the time of the previous study, the software for evaluating the collected GPL_IPL measurements was not yet developed. Now being able to study the GCL_IPL is advantageous as its topography shows a more direct relation between structure and function and in that sense predicts the VF defects in a simpler way. Most previous studies have used OCT of the RNFL, but OCT of the macular GCL_IPL is believed to be as stable, if not more so, and should be used for questions concerning the VF function. Reliable OCT measurements of the retina can be done already in the neonate (35), and thus, newborns with retinal thinning due to a presumed lesion to the posterior visual pathways could then be identified and prompted for an MRI for confirmation.

Given the difficulty of determining whether or not WMDI involves the OR, the OCT information could be very valuable. An obvious example is Subject G who suffered bilateral intra-ventricular hemorrhages and a right-sided periventricular hemorrhagic infarct (Figure 2C), who, despite this large injury, have normal appearing ORs on fiber tractography, normal GCL_IPL thickness and no VF defects. The combined information from OCT and fiber tractography explains her unaffected primary visual pathways, despite the suspicion of WMDI involvement of the OR on the structural MRI (36).

Although more pronounced in the superior sectors, the inferior GCL_IPL thickness was also reduced when comparing cases and controls. In some of the patients, it is likely that the brain injury not only involves the superior portion of the OR but to some extent also its inferior portion, resulting in subnormal inferior GCL_IPL thickness. For this reason, it seems important to study the GCL_IPL symmetry/asymmetry. This was done by using the ratio of the average GCL_IPL thickness in superior and inferior sectors, a parameter that clearly mirrored both the VF function and injury to the superior OR in relation to the inferior.

### Limitations

The small sample in this study is a limiting factor. Nevertheless, by thorough investigation of the seven patients, we were able to confirm the hypothesis of RTSD in the immature visual system in these individuals. The results are in line with previous studies on RTSD in humans (23, 24), but no one has previously shown this strict topographical and quantitative relationship between injuries to the immature geniculo-striate pathways and the secondary retinal changes. However, by including more cases, this correspondence could be further confirmed. The associations between GCL_IPL thinning and the OR injuries could be further characterized with additional evaluation of the dMRI data, as discussed above.

The patient group put a relative limitation on the task compliancy and for the processing of the retinotopic fMRI data. First, some patients had eye motility problems and difficulty with gaze stability, which affects fixation. Even though the noise levels in the fMRI signal were large, possibly due to compliance problems, the signal was stable and, given the fact that cortical VF maps even in these subjects appeared normal and did not differ from those of controls, we are confident that V1-ROIs represent the cortical V1-activation from the retinotopic stimulus. Similarly, none of the patients showed fixation problems during the VF examinations, indicating compliance problems rather than fixating problems in the retinotopic fMRI. Second, the gray/white matter segmentation step in the cortical flattening was difficult due to the WMDI lesions. Many patients had enlarged, irregular ventricles and periventricular gliosis. The 3D-T1w sequence could have been optimized to increase the gray/white matter contrast to ameliorate segmentation, and the processing step further improved to deal with lesions, e.g., by including segmentation masks.

The patient group is not representative of all individuals with WMDI as they were selected for their ability to cooperate in the demanding tests. Individuals with more extensive brain damage have more pronounced functional deficits, such as severe cerebral palsy, intellectual and attention problems, severe cerebral visual impairment, and eye motility disorders, disabling them to participate. However, there is no reason to believe that neither the GCL_IPL nor the VF function would be spared in individuals with more extensive WMDI. Nevertheless, and as shown in this study, individuals with extensive WMDI can have normal GCL_IPL thickness and normal VF function given that the injury has spared the primary visual pathways.

## Conclusion

The results of this study suggest the existence of a strict topographical relationship between injury to the superior parts of OR in WMDI and the thinning of the macular ganglion cell complex above the horizontal meridian, consistent with the extent of the inferior VF defects. Structural morphological changes were seen in the periventricular OR, with displacement of dorsal striate projections to the space occupied by ventral striate projections in the affected individuals. The severity of the primary injury in the OR, assessed by dMRI, is correlated with secondary neurodegeneration in the macular ganglion cell complex. Together, these results provide convincing evidence for RTSD in the immature human retinal and thalamocortical visual pathways and suggest a compensatory re-organization in the OR.

## Ethics Statement

This study was approved by the local ethics committee (EPN Stockholm Nord, diarienr 2013/1114-31/2). All subjects gave written informed consent in accordance with the Declaration of Helsinki.

## Author Contributions

Authorship credit was based on substantial contributions to (a) the conception and manuscript design (FL, MN, OF, LJ, and JL); (b) acquisition and analysis of data or interpretation of data (FL, MN, LJ, and JL); (c) drafting the article or revising it critically for important intellectual content (FL, MN, LJ, and JL); (d) final approval of the version to be published (FL, MN, OF, LJ, and JL); and (e) are accountable for all aspects of the work in ensuring that questions related to the accuracy or integrity of any part of the work are appropriately investigated and resolved (FL, MN, OF, LJ, and JL).

## Conflict of Interest Statement

None of the authors have any conflict of interest, including specific commercial or financial interests, relationships, or affiliations, relevant to the manuscript that could be construed as a potential conflict of interest.

## Footnotes

^1^http://gru.stanford.edu/doku.php/mrTools/overview (Accessed: April 26, 2018).

^2^http://www.pc.rhul.ac.uk/staff/J.Larsson/software.html (Accessed: April 26, 2018).

^3^https://github.com/MRtrix3/mrtrix3 (Accessed: April 26, 2018).

## Abbreviations

FA, fractional anisotropy; MD, mean diffusivity; D_//_, axial diffusivity; D_⊥_, radial diffusivity; AFD, Apparent fiber density; OR, optic radiation; OCT, optical coherence tomography; GCL_IPL, macular ganglion cell complex, including the ganglion cell layer and the inner plexiform layers; WMDI, white-matter damage of immaturity; VF, visual field; RNFL, retinal nerve fiber layer; dMRI, diffusion-weighted MRI; FOD, fiber orientation distributions; DTI, diffusion tensor imaging.
