# Supplementary material for: Injuries to the Immature Optic Radiation Show Correlated Thinning of the Macular Ganglion Cell Layer
Source: Front Neurol. 2018 May 7;9:321. doi: 10.3389/fneur.2018.00321 (PMC5950728; doi:10.3389/fneur.2018.00321)
Supplement: Supplementary file 1 [file Image_1.PDF]

The visual fields in the patient group

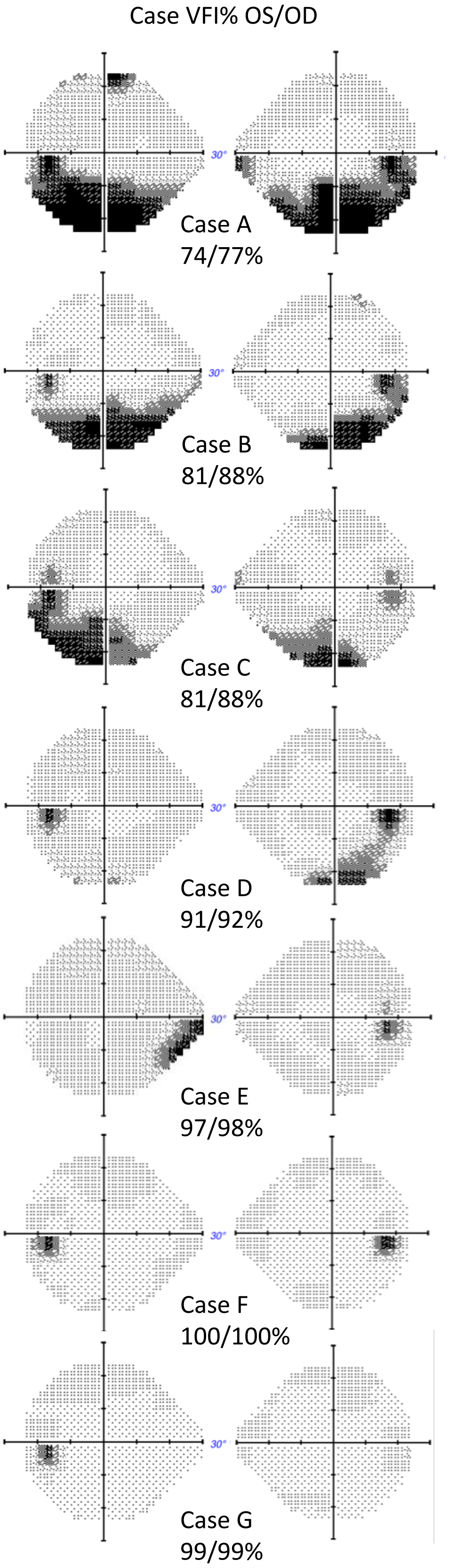

OS/OD=left/right eye. Central visual fields evaluated with Humphrey Field Analyser with SITA Fast 24-2. Reduced sensitivity indicated by dark colors in the gray scale maps. The visual field index (VFI) is a global index indicating the visual field function and 100% is associated with normal vision. The illustrations show varying extents of inferior visual field defects for Cases A-G mainly located in the inferior hemifields, which is typical in this patient group.
